# Enhancing cancer vaccine efficacy via electrostatic engineering of an FcγR-targeted protein

**DOI:** 10.3389/fimmu.2026.1831652

**Published:** 2026-05-22

**Authors:** Chiao-Chieh Wu, Chia-Ling Chen, Chen-Yi Chiang, Ling-Ling Tu, Shih-Jen Liu, Chih-Hsiang Leng, Hisn-Wei Chen

**Affiliations:** 1 National Institute of Infectious Diseases and Vaccinology, National Health Research Institutes, Zhunan, Miaoli, Taiwan; 2 Graduate Institute of Biomedical Sciences, China Medical University, Taichung, Taiwan; 3 Graduate Institute of Medicine, Kaohsiung Medical University, Kaohsiung, Taiwan

**Keywords:** antitumor efficacy, cancer vaccines, FLIPr, immunogenicity, immunotherapy

## Abstract

**Background:**

Effective cancer vaccines require delivery platforms that can simultaneously enhance immune responses, maintain safety, and simplify the formulation process. This study aimed to develop a novel vaccine delivery system based on the *Staphylococcus aureus*-derived formyl peptide receptor-like 1 inhibitory protein (FLIPr), which naturally targets Fcγ receptors on antigen-presenting cells.

**Methods:**

A recombinant variant, rF9R, was engineered by adding nine arginine residues to the C-terminus of FLIPr to facilitate electrostatic binding with anionic components. To optimize this interaction, epitopes were modified with five aspartic acid residues. The platform’s ability to form stable complexes with peptides, CpG oligodeoxynucleotides, and protein antigens was evaluated. The efficacy of the rF9R/rE7m complex was subsequently tested in tumor models to assess CD8^+^ T-cell activation and tumor regression.

**Results:**

The rF9R protein successfully bound peptides and antigens to form stable complexes, significantly enhancing antigen delivery and immune activation. *In vivo* results demonstrated that the rF9R/rE7m complex—both as a standalone treatment and when combined with CpG—elicited robust CD8^+^ T-cell responses. This immune activation led to significant tumor regression in the studied models.

**Conclusion:**

The rF9R platform functions as both an efficient carrier and a potent immunostimulatory component. By providing a simple and versatile method for delivering peptide and subunit vaccines, rF9R represents a promising strategy for advancing cancer immunotherapy.

## 1 Introduction

Cancer remains a major global health issue, causing millions of deaths annually despite significant advancements in medical research and treatments (1, 2). Over the past four decades, personalized cancer vaccines have emerged as a promising form of immunotherapy, designed to activate the immune system to recognize and eliminate tumor cells (3). Several cancer vaccines, including Bacillus Calmette-Guérin (BCG), Talimogene laherparepvec (TVEC), and Provenge (Sipuleucel-T), have been approved by the U.S. Food and Drug Administration (FDA) for clinical use (4).

While progress in identifying immunogenic cancer epitopes and developing vaccine technology has shown promise in phase 2b clinical trials (5), neoantigen-based immunotherapies still face challenges in achieving consistent clinical efficacy (6). Key challenges include immune suppression within the tumor microenvironment, insufficient T-cell responses, suboptimal vaccine formulation and delivery, inadequate adjuvant selection, and the inherent diversity of tumor types (7). Overcoming these barriers is essential for advancing cancer vaccines and improving treatment outcomes. Researchers are exploring innovative strategies, such as targeting tumor antigens to antigen-presenting cells (APCs), enhancing tumor antigen expression, employing epigenetic remodeling, and inducing immunogenic cell death (8).

Unlike infectious diseases, where pathogens are easily recognized as foreign, cancer cells originate from the body’s own tissues. This endogenous nature results in tumor antigens exhibiting weak immunogenicity, often failing to trigger a strong immune response. Because cancer cells closely resemble normal cells, the immune system struggles to distinguish and effectively target them (9). Immunogenicity is a critical factor in therapeutic cancer vaccines, as it determines the strength and quality of the immune response needed to suppress tumor growth and establish long-term antitumor immunity. These vaccines work by activating the adaptive immune system, delivering tumor antigens to dendritic cells (DCs), which then process and present them to CD4^+^ helper T cells and CD8^+^ cytotoxic T cells - both essential for identifying and eliminating tumor cells (10). To maximize the therapeutic potential of neoantigen cancer vaccines, overcoming the weak immunogenicity of tumor antigens is critical (6). However, the development of these vaccines has been hindered by the lack of simple, safe, and broadly effective adjuvants. Addressing this challenge is essential to advance cancer immunotherapy and facilitate widespread clinical adoption.

Our previous research demonstrated that formyl peptide receptor-like 1 inhibitory protein (FLIPr) can serve as an effective vehicle for antigen delivery. Fusion of antigens to FLIPr enabled efficient presentation via both MHC class I and II pathways, resulting in robust and long-lasting memory T-cell responses (11, 12). Therefore, we expand on previous research, further validating FLIPr’s role in cancer immunotherapy and demonstrating its potential to streamline vaccine design, enhance safety, and boost immunogenicity.

This study introduces rF9R, a genetically engineered protein generated by adding nine arginine residues to the C-terminus of FLIPr, as a novel platform to enhance therapeutic cancer vaccines. The arginine-rich modification strengthens electrostatic interactions, enabling robust binding to negatively charged vaccine components and potentially overcoming the low immunogenicity of tumor antigens. Preclinical studies have shown that rF9R, when combined with a non-oncolytic mutant HPV E7 protein (rE7m), induces potent antitumor immunity (13), while pairing with CpG oligodeoxynucleotides activates innate immune responses via Toll-like receptor 9 (TLR9) signaling (14). Building on these findings, we hypothesize that rF9R functions as a dual-purpose platform that enhances both antigen delivery and immune activation, thereby improving the immunogenicity and therapeutic efficacy of cancer vaccines. To test this, we evaluate the ability of rF9R-based formulations to promote antigen presentation, dendritic cell activation, and antitumor immune responses in preclinical tumor models, aiming to establish rF9R as a versatile strategy for overcoming current limitations in cancer vaccine development.

## 2 Materials and methods

### 2.1 Ethics statement

All animal studies were conducted in full compliance with Taiwan’s Animal Protection Act. The study protocols were approved by the Animal Committee of the National Health Research Institutes (Protocol No: NHRI-IACUC-108157-A) and carried out in accordance with institutional guidelines, with full adherence to the ARRIVE guidelines. Mice were anesthetized with 2–4% isoflurane for initiation and 0.5–2.0% for maintenance, or euthanized by gradual (30–70% per minute) displacement of chamber air with compressed CO_2_.

### 2.2 Production and characterization of recombinant FLIPr and recombinant F9R

The construction of rFLIPr expression vectors has been previously described (15). The DNA sequence of FLIPr (accession number BAB57318) was synthesized by Purigo Biotechnology Co. (Taipei, Taiwan) using *Escherichia coli* codon optimization based on the amino acid sequence of FLIPr. The plasmid pF9R, inserted into the pET-28a (+) vector, was ordered and constructed by TrustGene Biotech Company Limited (Taichung, Taiwan). The pF9R plasmid was transformed into *Escherichia coli* BLR (DE3) (Novagen), and recombinant F9R (rF9R) expression was induced with 1 mM isopropyl-β-D-thiogalactopyranoside (IPTG). Harvested bacterial cells were resuspended in lysis buffer (20 mM Tris, pH 8.9, containing 0.5 M NaCl) and disrupted using a French press (Constant Systems, Daventry, UK) at 27 kpsi. The clarified cell lysate was subjected to purification using an immobilized metal affinity chromatography (IMAC) column (QIAGEN, Ni-NTA Superflow). The column was sequentially washed with 10 column volumes of wash buffer (lysis buffer containing 20 mM imidazole and subsequently 40 mM imidazole). rF9R was then eluted using three column volumes of elution buffer (1X PBS, 0.5 M NaCl, 500 mM imidazole, pH 7.5). To remove residual imidazole and prepare the protein for downstream assays, the purified rF9R was subjected to a two-stage dialysis process. Initially, the protein was dialyzed against 20 mM sodium phosphate (pH 5.8). Following the removal of any minor precipitate via centrifugation, the sample underwent three subsequent buffer changes against 10 mM ammonium acetate (pH 5.5). Each dialysis step was conducted for a minimum of 6 hours at 4 °C to ensure the exhaustive removal of imidazole and optimal protein stability. To ensure the absence of significant endotoxin contamination, the residual lipopolysaccharide (LPS) content in the purified rF9R was quantified using a Limulus amebocyte lysate (LAL) assay (Associates of Cape Cod, Inc., Cape Cod, MA, USA) according to the manufacturer’s instructions. The endotoxin levels were determined to be less than 30 EU/mg, confirming that the preparation was suitable for *in vivo* immunological assays. The purified rF9R was analyzed by 10% SDS-PAGE and immunoblotting.

### 2.3 Measurement of binding efficacy in rFLIPr and rF9R

The biotinylation of rFLIPr and rF9R was performed using the EZ-Link NHS-PEG4-Biotinylation Kit (Thermo Fisher Scientific, Rockford, IL, USA) according to the manufacturer’s instructions. Fcγ receptors were obtained from Sino Biological, Inc. (Chesterbrook, PA, USA) or ACROBiosystems (Newark, DE, USA). Mouse Fcγ receptors (FcγR-1, -2b, -3, and -4) and human Fcγ receptors (FcγR-1, -2b, -2a [H167], -2a [R167], -3a [F176], -3a [V176], -3b [NA1], and -3b [NA2]) were coated onto 96-well plates at 0.2 µg/well. To prevent nonspecific binding, wells were blocked with 5% skim milk in PBS. A serial 3-fold dilution of biotin-conjugated rE7m, rFLIPr, and rF9R (starting at 333 nM) was added to the wells and incubated at room temperature for 2 hours. After washing, HRP-conjugated streptavidin (Biolegend, CA) was added to detect bound proteins. The reaction was developed using 3,3’,5,5’-tetramethylbenzidine (TMB) (CLINiCAL, US) as a substrate and was incubated for 10 minutes. Absorbance was measured at 450 nm using an ELISA reader.

### 2.4 Electrophoretic mobility shift assay for detecting the association of rFLIPr and rF9R with antigens

An electrophoretic mobility shift assay (EMSA) was conducted to confirm the binding of rFLIPr and rF9R to peptides or rE7m. Purified rFLIPr and rF9R (0 - 36 μM) were incubated in 1X PBS (pH 7.4) at 25 °C for 30 minutes. The reaction mixtures were then combined with 2X sample buffer (62.5 mM Tris, 40% glycerol, 0.01% bromophenol blue sodium salt, pH 6.8) and loaded onto a 12% native polyacrylamide gel. Electrophoresis was performed at 100 V and 25 °C for 110 minutes in 1X Tris/Glycine running buffer. After electrophoresis, the gel was stained with Coomassie Brilliant Blue (SIGMA-ALDRICH, US) to visualize protein bands. To investigate the interaction between CpG (ODN 1826: TCCATGACGTTCCTGACGTT) (GeneDireX, TW) and rFLIPr or rF9R, CpG (6 μM) was incubated with two-fold serial dilutions of rFLIPr (0.75–6 μM) or rF9R (0.75–6 μM) in 1X PBS (pH 7.4) at 25 °C for 30 minutes. The reaction mixtures were then combined with 6X loading buffer (60% glycerol in sterile water) and loaded onto a 1% native agarose gel. Electrophoresis was carried out at 100 V and 25 °C for 20 minutes in running buffer (43 mM imidazole, 35 mM HEPES), followed by staining with DNA RedSafeTM Nucleic Acid Staining Solution (iNtRON, KR) and Coomassie Blue.

### 2.5 ELISA for the detection of carrier/CpG complexes

An ELISA was performed to validate the binding interaction between the carriers and CpG (ODN 1826). Costar assay plates (Corning) were coated overnight at 4 °C with 100 µl of either rFLIPr or rF9R at a concentration of 0.1 mg/ml in 0.1 M Na_2_CO_3_ coating buffer (pH 9.5). Wells coated with buffer alone (without rFLIPr or rF9R) served as negative controls. Following coating, the plates were washed three times with PBS containing 0.05% (v/v) Tween 20 and then blocked with 5% (w/v) non-fat milk in PBS for 1 hour at room temperature. After blocking, the wells were incubated with a serial dilution of biotinylated CpG (ODN 1826) prepared in PBS containing 0.05% (v/v) Tween 20 and 5% (w/v) non-fat milk. The incubation was carried out for 2 hours at room temperature. Afterward, the plates were washed three times and incubated for 90 minutes with HRP-conjugated streptavidin to detect bound biotinylated CpG. To develop the signal, 100 µl of TMB substrate was added per well and incubated with shaking (200 rpm) for 15 minutes in the dark. The reaction was stopped by adding 100 µl of 2N H_2_SO_4_ per well, and absorbance was measured at 450 nm using an ELISA plate reader.

### 2.6 RAW264.7 macrophage cell activation assay

To assess macrophage activation, RAW264.7 cells were cultured in RPMI-1640 medium supplemented with 10% fetal bovine serum (FBS) and 1% penicillin-streptomycin. Cells were maintained at 37 °C in a 5% CO_2_ incubator. Cells were seeded into 24-well plates at a density of 2 × 10^5^ cells per well and incubated overnight to allow adherence. The next day, cells were treated with varying concentrations of CpG, rFLIPr, rF9R, CpG/rFLIPr, or CpG/rF9R for 24 hours. Following treatment, cells were harvested and stained with fluorophore-conjugated antibodies against CD40 (BD Pharmingen, US) (1:100 dilution), CD80 (Biolenged, CA) (1:100 dilution), and CD86 (Invitrogen, CA) (1:100 dilution). Flow cytometry was performed to evaluate the surface expression of these co-stimulatory molecules. Data were analyzed using FlowJo software (Attune NxT, Ivitrogen), and activation levels were compared across treatment groups.

### 2.7 Internalization of CpG/F9R into RAW 264.7 cells and its association with TLR9

To evaluate the cellular internalization of the CpG/rF9R complex and its colocalization with the TLR9 receptor, confocal microscopy was performed using RAW264.7 macrophage-like cells. Cells were seeded onto glass coverslips in 24-well plates at a density of 1 × 10^5^ cells per well and incubated overnight at 37 °C in a 5% CO_2_ atmosphere. The following day, cells were treated with PBS, rF9R, CpG-biotin, or CpG/rF9R complex for 4 hours. After treatment, cells were washed with PBS and fixed with 4% paraformaldehyde for 15 minutes at room temperature. Fixed cells were permeabilized with 0.1% Triton X-100 for 10 minutes, followed by blocking with 1% BSA (SIGMA-ALDRICH, US) in PBS for 30 minutes to prevent nonspecific binding.

Cells were then incubated with a TLR9 antibody conjugated with Alexa Fluor^®^ 488 (1:50 dilution) (Novus, US) for overnight at 4 °C. CpG internalization was visualized using streptavidin-Alexa Fluor 594 (Invitrogen, CA), and nuclei were counterstained with Hoechst 33342 (Invitrogen, CA). Images were acquired using a confocal laser scanning microscope. Colocalization of the CpG/rF9R complex with TLR9 was analyzed using ImageJ software.

### 2.8 *In vivo* tumor model studies

TC-1 Tumor Model: TC-1, a mouse epithelial cell line transformed with the oncogenes Ras, HPV16 E6 and E7, was a kind gift from Dr. T-C Wu (Johns Hopkins University). Female, 6- to 8-week-old naïve C57BL/6 mice were subcutaneously implanted with TC-1 cells (2 × 10^5^ cells per mouse) into the left flank before undergoing chemotherapy or immunization. To evaluate the antitumor effects of rF9R complex treatment, tumor-bearing mice were randomly assigned to groups. After tumor implantation, mice in the peptide-based groups received subcutaneous (s.c.) injections on days 3 and 10, while those in the rF9R/protein complex groups were immunized on days 7 and 14. All treatments (100 µL in PBS) were administered into the dorsum.

EG7 Tumor Model: EG7 cells (BCRC #60418, Bioresource Collection and Research Center, Hsinchu, Taiwan) are derived from EL4, a mouse lymphoma cell line, and stably transfected with the OVA gene to constitutively produce OVA. Female, 6- to 8-week-old naïve C57BL/6 mice were subcutaneously implanted with 2 × 10^5^ EG7 cells in the left flank. Once tumors were established (3 days post-tumor inoculation), mice were randomly divided into groups to assess the antitumor efficacy of rF9R complexes. Each group received a subcutaneous (s.c.) injection of rF9R complexes (100 µL PBS) into the dorsum. Tumor progression and treatment responses were closely monitored throughout the study.

Tumor diameters were measured with a caliper, and tumor volume (V) was calculated according to the formula: V = width × length × (width + length)/2. The percentage of tumor growth inhibition (TGI) was determined using the following equation: TGI (%) = [1 - (tumor volume in the treatment group/tumor volume in the PBS group)] × 100%.

### 2.9 ELISPOT assay

To evaluate the antigen-specific cellular immune response elicited by rF9R complex-based formulations, an ELISPOT assay (BD, US) was conducted to quantify IFN-γ-secreting CD8^+^ T cells. C57BL/6 mice were immunized twice at a two-week interval with one of the following formulations: PBS, rE7m, rE7m/rF9R, CpG/rE7m, or CpG/rF9R + rE7m/rF9R. Seven days after the final immunization, spleens were collected under sterile conditions. Splenocytes were isolated through mechanical dissociation and filtration using a 70-μm cell strainer. Red blood cells were lysed with ammonium–chloride–potassium (ACK) lysing buffer (Biolegend, CA), and the remaining splenocytes were washed and resuspended in RPMI 1640 medium supplemented with 10% FBS.

For the ELISPOT assay, 96-well PVDF plates (Millipore; Cork, Ireland) were pre-coated overnight at 4 °C with anti-mouse IFN-γ capture antibody (5 μg/mL). Plates were then washed with PBS and blocked with RPMI 1640 containing 10% FBS for 1 hour at 37 °C. Splenocytes (2 × 10^5^ cells per well) were plated and stimulated for 48 hours at 37 °C in 5% CO_2_ with either the HPV E7_49–57_ peptide (RAHYNIVTF, 10 μg/mL) or the control OVA_257–264_ peptide (SIINFEKL). After incubation, wells were washed and incubated with biotinylated anti-IFN-γ detection antibody (2 μg/mL) for 2 hours at room temperature, followed by streptavidin-HRP (1:1000 dilution) (BD, US) for 1 hour. Spots were developed using angiotensin-converting enzyme (ACE) substrate (SIGMA-ALDRICH, US) and the reaction was stopped with distilled water. IFN-γ-producing cells were visualized and quantified using an automated ELISPOT reader (Cellular Technology, US). Results were expressed as the number of spot-forming units (SFUs) per 10^6^ splenocytes.

### 2.10 Statistical analysis

The statistical significance of differences between mean values of the experimental groups was determined using the Kruskal–Wallis test with Dunn’s multiple comparison test. *P < 0.05, **P < 0.01, ***P < 0.001,and ****P < 0.0001.

## 3 Results

### 3.1 Production and characterization of rFLIPr and rF9R

**Figure 1:**
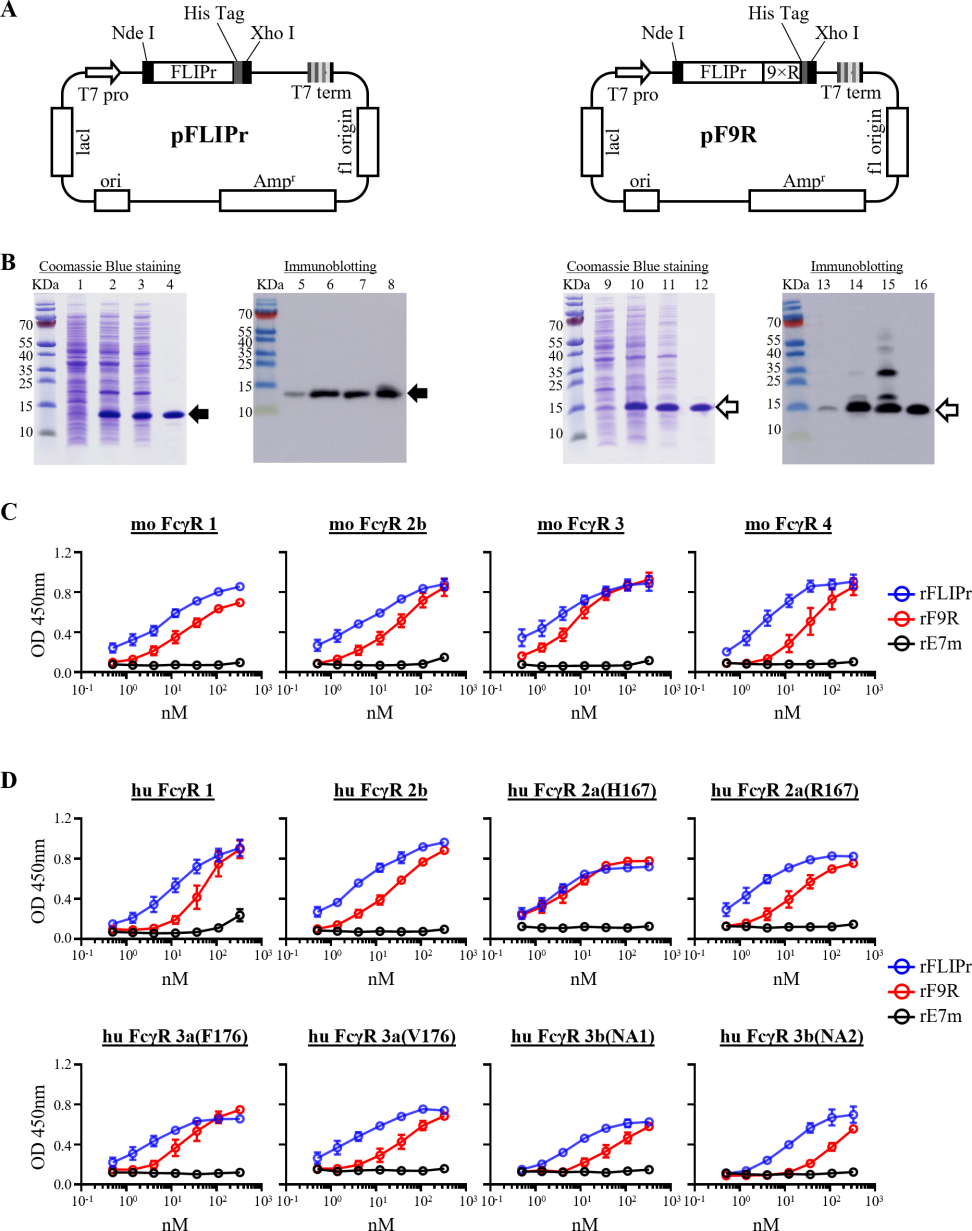
Expression, purification, and Fcγ receptor binding of recombinant FLIPr (rFLIPr) and F9R (rF9R). **(A)** Plasmid maps illustrating constructs encoding rFLIPr (pFLIPr) and rF9R (pF9R). **(B)** rFLIPr and rF9R were expressed in *E. coli* BL21 (DE3) and BLR (DE3), respectively, and purified using immobilized metal affinity chromatography (IMAC). Expression and purification were analyzed by 10% reducing tricine SDS-PAGE followed by Coomassie blue staining (lanes 1–4 for rFLIPr; 9–12 for rF9R) and immunoblotting with anti-HisTag antibodies (lanes 5–8 for rFLIPr; 13–16 for rF9R). Lane 1, 5, 9 and 13, total lysate without IPTG induction; Lane 2, 6, 10 and 14, lysate after IPTG induction; Lane 3, 7, 11, and 15, soluble fraction; Lane 4, 8, 12, and 16, purified proteins. Solid arrows mark rFLIPr, and open arrows mark rF9R. **(C, D)** Fcγ receptor binding was evaluated by ELISA using biotin-labeled rFLIPr and rF9R, with rE7m as a negative control. Immobilized mouse **(C)** or human **(D)** Fcγ receptors (0.5 μg/well) were incubated with protein dilutions. Binding was detected by HRP-streptavidin and TMB, with absorbance read at 450 nm. Statistical analysis was performed using two-way ANOVA. For all tested variants, significant binding (*P* < 0.05) was observed starting from low to mid-nanomolar concentrations (ranging from 0.5 nM to 37 nM) up to 333.3 nM, compared to the negative control.

The production of recombinant FLIPr (rFLIPr) was performed as previously described (15). F9R were cloned into the pET-28a (+) vector under the control of the T7 promoter, with a C-terminal six-histidine (HisTag) sequence to facilitate purification. A schematic representation of these plasmids is shown in **Figure 1A**. The resulting plasmid (pF9R) was transformed into *E. coli* BLR (DE3), and protein expression was induced with isopropyl β-D-thiogalactoside (IPTG). Lysates were subjected to immobilized metal affinity chromatography (IMAC), yielding purified rF9R. SDS-PAGE with Coomassie blue staining and immunoblotting using anti-HisTag antibodies confirmed the purity and expected molecular weights of rFLIPr (~14 kDa) and rF9R (~15 kDa) (**Figure 1B**). To minimize immunogenic interference, lipopolysaccharide (LPS) levels in the final preparations were reduced to <30 EU/mg.

Given the C-terminal modification of rF9R, we evaluated whether this change affected its biological activity. Binding to mouse Fcγ receptors (FcγR1, FcγR2b, FcγR3, and FcγR4) was assessed using ELISA, with rFLIPr and rE7m as positive and negative controls, respectively. The half-maximal effective concentration (EC_50_) values of rF9R were 18 nM (FcγR1), 30 nM (FcγR2b), 9 nM (FcγR3), and 30 nM (FcγR4), compared to 9, 10, 5.5, and 4 nM for rFLIPr (**Figure 1C**). Similarly, we tested human Fcγ receptors, including FcγR1, FcγR2a (H167 and R167), FcγR2b, FcγR3a (F176 and V176), and FcγR3b (NA1 and NA2). rF9R demonstrated effective binding to all eight subtypes with EC_50_s ranging from 5 to 80 nM - consistently higher than those of rFLIPr (**Figure 1D**). These results confirm that rF9R maintains FcγR-binding capacity despite its structural modification, supporting its potential use as an immune-targeting platform.

### 3.2 rF9R forms stable complexes with anionic peptides and enhances antitumor efficacy in EG7 and TC-1 tumor models

To test its electrostatic binding capacity, rF9R was evaluated for complexation with synthetic OVA peptides in the EG7 tumor model. Four peptides were tested: LOT1 (OVA_254–269_), 5D-LOT1 (LOT1 plus five N-terminal aspartic acids), LOT2 (OVA_320–345_), and 5D-LOT2 (LOT2 plus five aspartic acids).

**Figure 2:**
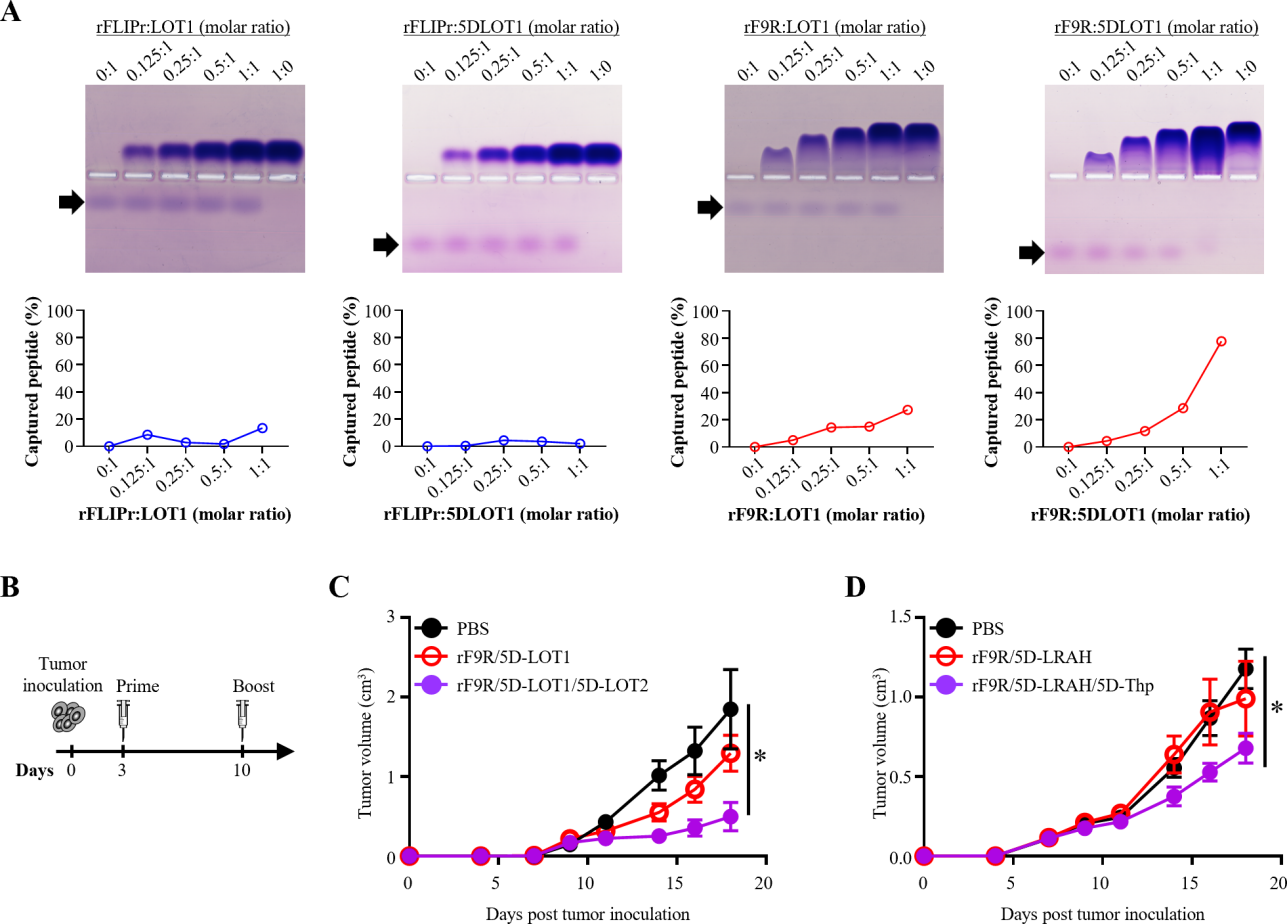
rF9R stably binds anionic peptides and enhances antitumor activity in EG7 and TC-1 models. **(A)** EMSAs were performed to evaluate the binding between rFLIPr or rF9R and synthetic peptides (LOT1, 5D-LOT1) at increasing molar ratios (0:1 to 1:1). A 1:0 protein-only lane served as control. Native PAGE (upper panels) and peptide capture quantification (lower panels) are shown. Arrows indicate free peptides. **(B)** Immunization schedule: mice (n = 5) received tumor cells on day 0, followed by primary (day 3) and booster (day 10) immunizations with peptide–protein complexes. **(C)** Tumor growth in EG7-bearing mice treated with PBS, rF9R/5D-LOT1, or rF9R/5D-LOT1/5D-LOT2. Dual-peptide treatment delayed tumor progression. **(D)** Tumor growth in TC-1-bearing mice treated with PBS, rF9R/5D-LRAH, or rF9R/5D-LRAH/5D-Thp. Combination of CTL and helper peptides reduced tumor volume. Statistical analysis was performed using the Kruskal–Wallis test followed by Dunn’s multiple comparison test for the terminal time points. *P < 0.05.

Electrophoretic mobility shift assays (EMSAs) were used to assess complex formation. Peptide binding was tested across molar ratios of carrier:peptide from 0:1, 0.125:1, 0.25:1, 0.5:1 to 1:1. The 1:0 ratio, containing only the carrier protein, served as the blank control. Unbound peptides appeared as distinct bands on the gel, with reduced intensity indicating complex formation (**Figure 2A**, upper panel). rF9R showed stronger interaction with 5D-LOT1 than with unmodified LOT1. Quantification revealed that while rFLIPr bound poorly to both peptides, rF9R achieved ~20% binding to LOT1 and >80% to 5D-LOT1 at the 1:1 ratio (**Figure 2A**, lower panel). These results indicate that rF9R preferentially binds to negatively charged peptides, and 5D modifications significantly enhance this interaction.

*In vivo* evaluation of antitumor efficacy was performed in EG7-bearing mice, treated on days 3 and 10 after tumor implantation (**Figure 2B**). The percentages of tumor growth inhibition on day 18 for the rF9R/5D-LOT1 and rF9R/5D-LOT1/5D-LOT2 groups were 29.9% and 73.2%, respectively. These results suggest a synergistic effect between cytotoxic and helper epitopes (**Figure 2C**).

To validate these findings in another model, we tested a similar approach in the TC-1 tumor system using 5D-modified HPV E7-derived peptides: 5D-LRAH (CTL epitope; E7_45–57_ (16)) and 5D-Thp (PADRE helper peptide (17)). As shown in **Figure 2D**, rF9R/5D-LRAH alone achieved 15.9% tumor growth inhibition on day 18, while co-formulation with 5D-Thp further enhanced the antitumor effects, resulting in 42.3% inhibition. These results reinforce the value of combining CTL and helper epitopes in rF9R-based peptide vaccines, supporting the use of rF9R as an effective carrier in peptide-based cancer vaccines.

### 3.3 rF9R enhances the antitumor efficacy of protein-based vaccines

**Figure 3:**
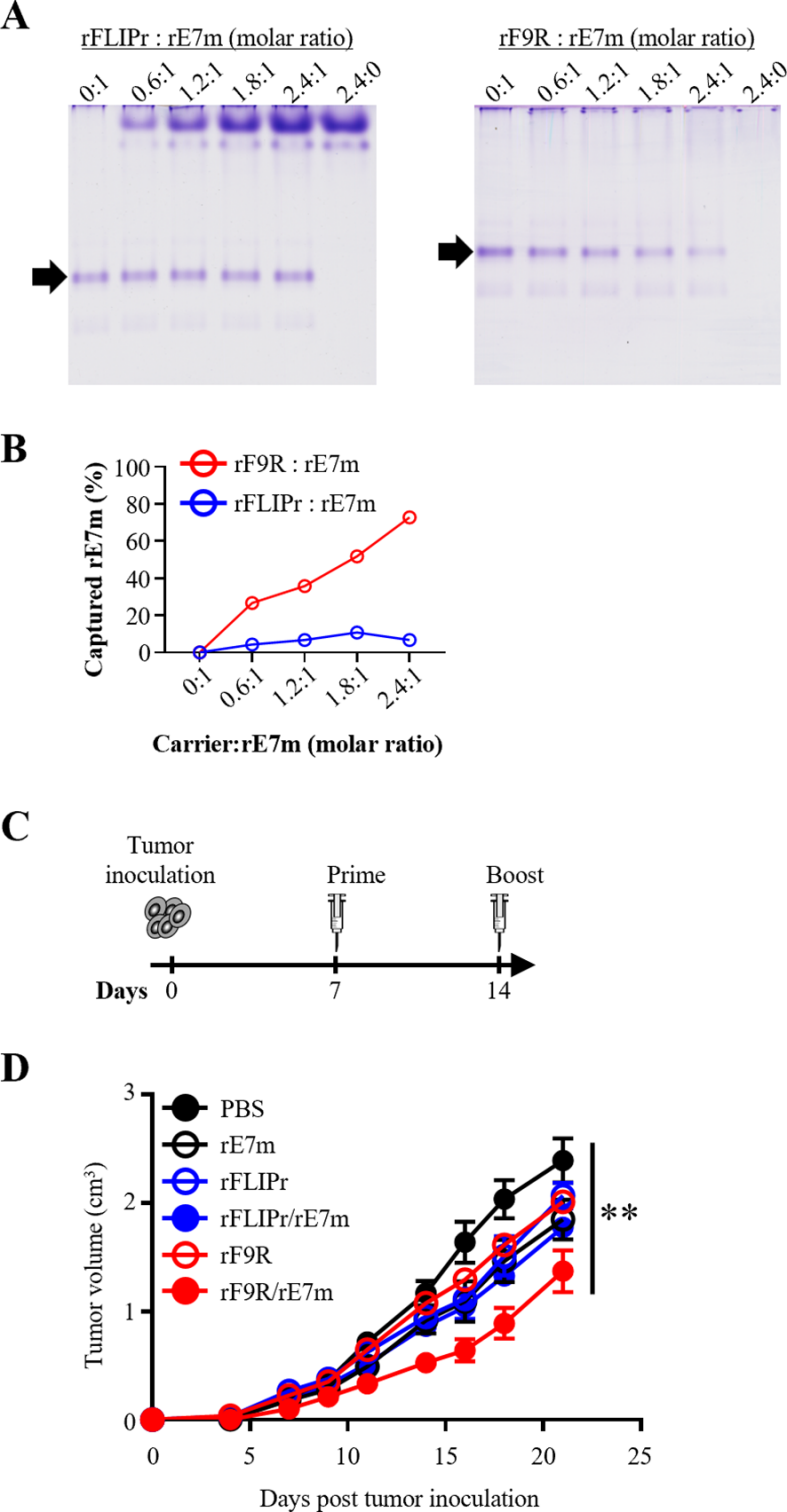
rF9R binds HPV16 E7 mutant protein (rE7m) and enhances therapeutic antitumor immunity. **(A)** Native PAGE of rE7m binding to rFLIPr or rF9R at various molar ratios of carrier:rE7m from 0:1, 0.6:1, 1.2:1, 1.8:1 to 2.4:1. The 2.4:0 protein-only control is included. Arrows indicate unbound rE7m. Strong shifts with rF9R suggest effective complex formation. **(B)** Quantification of rE7m capture efficiency showed dose-dependent binding by rF9R, minimal by rFLIPr. **(C)** Therapeutic schedule: tumor inoculation on day 0, followed by priming (day 7) and booster (day 14) with antigen–carrier complexes (n = 5). **(D)** Tumor growth curves comparing treatments (PBS, rE7m, rFLIPr, rFLIPr/rE7m, rF9R, rF9R/rE7m). rF9R/rE7m showed the strongest tumor suppression. Statistical analysis was performed using the Kruskal–Wallis test followed by Dunn’s multiple comparison test for the terminal time points. **P < 0.01.

To assess whether rF9R could serve as a carrier for full-length protein antigens, we examined its ability to bind rE7m, a recombinant HPV16 E7 mutant. Binding analysis revealed stronger rE7m interaction with rF9R than with rFLIPr in a dose-dependent manner (**Figures 3A, B**), confirming stable complex formation.

The rF9R/peptide complex contains only a single minimal CD8 epitope, whereas the rF9R/rE7m complex harbors multiple potential CD8 epitopes. Given this increased antigenic complexity and the potential for a more potent, multi-epitopic immune response, the rF9R/rE7m complex was evaluated in a more advanced tumor model (initiation on day 7) to demonstrate its superior therapeutic robustness (**Figure 3C**). Tumor growth inhibition was monitored over 20 days post-transplantation (**Figure 3D**). The percentage of tumor growth inhibition on day 21 in rE7m, rFLIPr, rFLIPr/rE7m, rF9R, and rF9R/rE7m were 22.8%, 13.6%, 16.0%, 25.8%, and 42.7%, respectively. Among these, the rF9R/rE7m group showed the strongest tumor suppression compared with all other groups, suggesting that the formation of an rF9R/rE7m complex could enhance the antitumor effect.

Collectively, these results support the potential of rF9R to enhance protein-based immunotherapies. Its ability to form stable complexes with negatively charged tumor antigens, such as rE7m, and to enhance therapeutic efficacy highlights rF9R as a promising candidate for the development of next-generation cancer vaccines.

### 3.4 rF9R efficiently binds CpG and enhances TLR9-mediated innate immune activation

**Figure 4:**
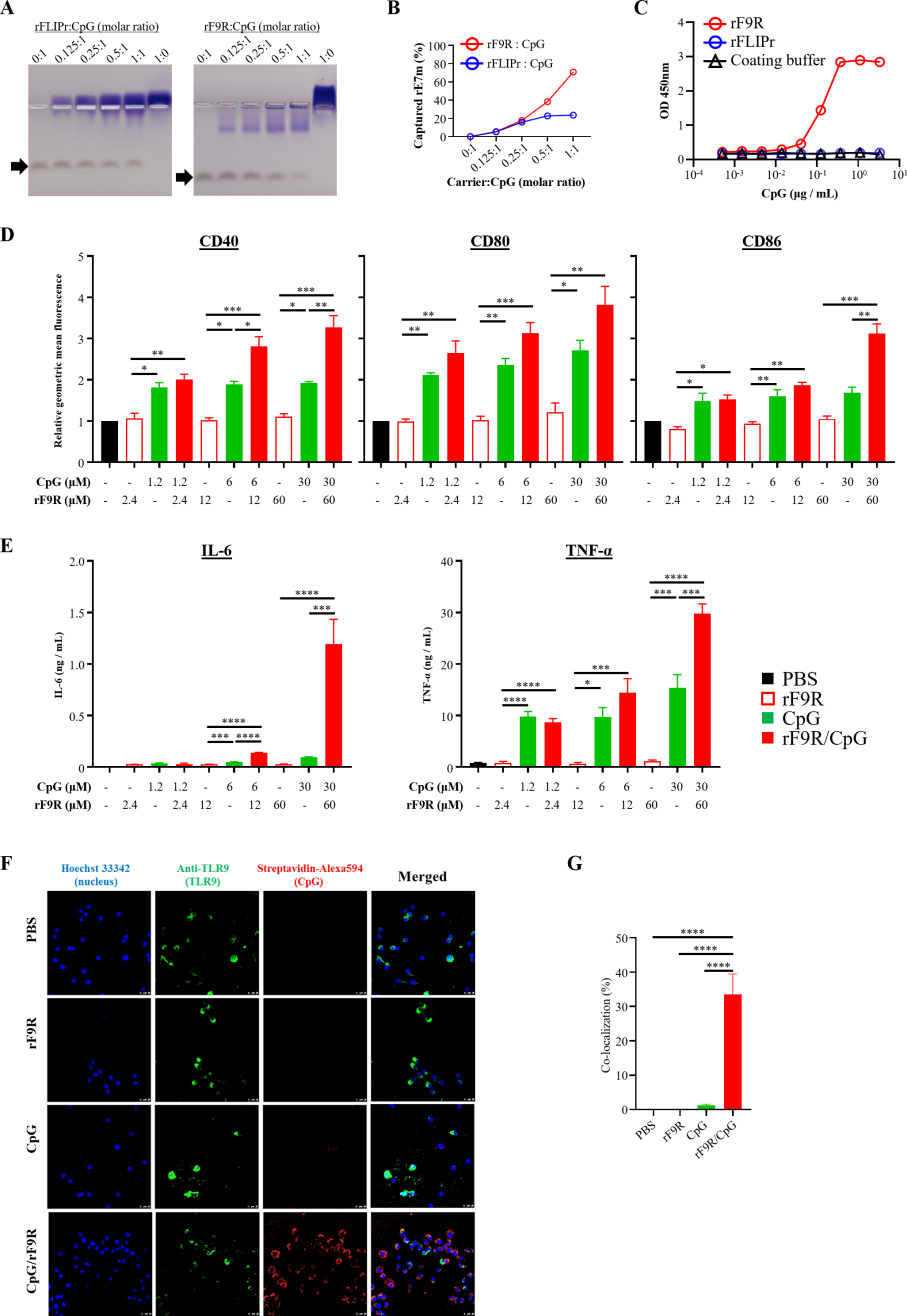
rF9R binds CpG and enhances macrophage activation via TLR9. **(A)** EMSA was used to assess CpG (6 μM) binding to rFLIPr or rF9R at protein:CpG molar ratios of 0:1, 0.125:1, 0.25:1, 0.5:1, and 1:1. A 1:0 ratio (protein only) served as the control. Samples were run on native agarose gels and sequentially stained with DNA SafeStain (CpG) and Coomassie (protein). Arrows indicate CpG bands. rF9R formed stronger complexes. **(B)** Quantification confirmed dose-dependent CpG binding by rF9R but not rFLIPr. **(C)** ELISA validated CpG interaction with rF9R; rFLIPr showed no binding. **(D)** RAW264.7 cells were treated with CpG, rF9R, or their complex for 24 hours, and the expression levels of CD40, CD80, and CD86 were analyzed by flow cytometry. **(E)** RAW264.7 cells were treated with CpG, rF9R, or their complex for 48 hours, and IL-6 and TNF-α levels were measured by ELISA. **(F)** Confocal microscopy of RAW264.7 cells treated with PBS, rF9R, CpG-biotin, or rF9R/CpG-biotin. Nuclei were stained with Hoechst (blue), TLR9 with green, and CpG with red. Merged images revealed strong CpG–TLR9 colocalization in the rF9R/CpG group. **(G)** Quantification confirmed the highest colocalization percentage with rF9R/CpG complexes (mean value ± SEM; n = 5), supporting enhanced TLR9 engagement. *P < 0.05, **P < 0.01, ***P < 0.001, ****P < 0.0001.

Immunopotentiators, such as the TLR9 agonist CpG (18) (a negatively charged oligodeoxynucleotide) can enhance antitumor activity when combined with tumor antigens. To investigate whether rF9R can bind CpG and synergistically boost immune responses, thereby enhancing the antitumor effects of rE7m, we conducted a binding assay to compare the interactions of CpG with rFLIPr and rF9R. As illustrated in **Figure 4A**, CpG exhibited stronger binding to rF9R than to rFLIPr. Quantitative analysis confirmed a dose-dependent increase in CpG binding to rF9R (**Figure 4B**), indicating that CpG mixed with rF9R could form a stable rF9R/CpG complex. Furthermore, a captured ELISA was performed to validate the binding interaction between the carriers and biotin conjugated-CpG. **Figure 4C** clearly showed that rF9R/CpG complex could be detected in a dose-dependent manner, whereas rFLIPr showed no detectable rFLIPr/CpG complex as coating buffer controls.

To assess the immunostimulatory effects of the CpG/rF9R complex, RAW264.7 murine macrophage-like cells were treated with various formulations, including CpG, rF9R, CpG and rF9R/CpG. Among these, the rF9R/CpG complex most effectively upregulated the co-stimulatory molecules CD40, CD80, and CD86 (**Figure 4D**) and induced the highest secretion levels of IL-6 and TNF-α at a concentration of 30 µM (**Figure 4E**). These results demonstrate significantly enhanced immune activation compared to the other treatments, highlighting the superior adjuvant potential of the rF9R-modified complex.

Since CpG binds to rF9R, we further analyzed the internalization of the rF9R/CpG complex in RAW264.7 cells and its association with the TLR9 receptor. Confocal microscopy was performed on cells incubated with PBS, rF9R, CpG, or the rF9R/CpG complex. As shown in **Figure 4F**, staining with Hoechst 33342 to reveal the nucleus, anti-TLR9 to observe theTLR9, and streptavidin-Alexa594 to detect CpG revealed colocalization of rF9R/CpG and TLR9 in the final merged column. **Figure 4G** presents the quantitative analysis, demonstrating that the rF9R/CpG complex exhibits the highest percentage of colocalization with TLR9, further supporting its role in immune activation.

These findings underscore the potential of the rF9R/CpG complex as a potent immunostimulatory agent, capable of promoting innate immune activation and enhancing antitumor responses through TLR9 signaling pathways.

### 3.5 rF9R complex-based vaccine elicits potent CD8^+^ T cell responses and suppresses tumor growth in a TC-1 mouse model

**Figure 5:**
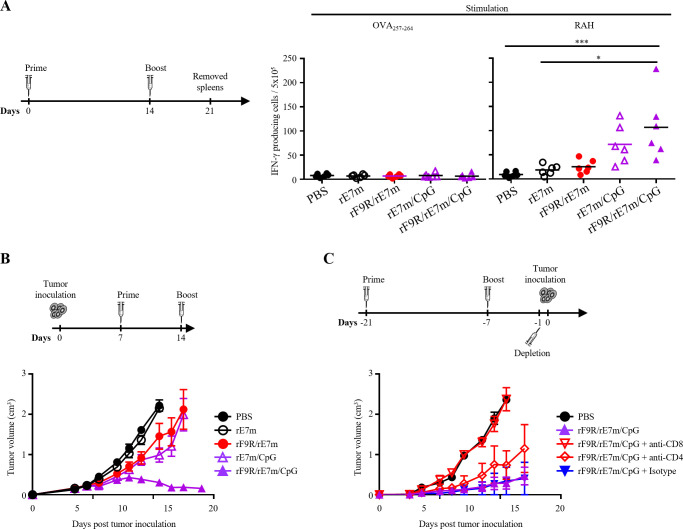
Combination of rF9R, rE7m and CpG elicits potent CD8^+^ T cell responses and promotes antitumor immunity in a TC-1 tumor model. **(A)** Mice (n = 6/group) were immunized subcutaneously on days 0 and 14 with PBS, rE7m, rF9R/rE7m, rE7m/CpG, or rF9R/rE7m/CpG. On day 21, splenocytes were harvested and restimulated *ex vivo* with HPV E7_49_–_57_ (RAH) or OVA_257_–_264_ (control). ELISPOT analysis of IFN-γ–secreting CD8^+^ T cells revealed significantly higher responses to RAH in the rF9R/rE7m/CpG group (*P < 0.05, ***P < 0.001). **(B)** Mice (n = 5/group) were inoculated with TC-1 cells (2 × 10_5_) on day 0 and treated on days 7 and 14. Tumor growth was monitored every 2–3 days. The rF9R/rE7m/CpG combination showed the most pronounced tumor suppression. **(C)** CD8^+^ T cell depletion abolished the antitumor effect of rF9R/rE7m/CpG, while CD4^+^ depletion had minimal impact. This confirms CD8^+^ T cells as key effectors in tumor control (n = 5/group).

To evaluate the immunogenicity of rF9R complex-based formulations, C57BL/6 mice were immunized twice at a two-week interval with PBS, rE7m, rF9R/rE7m, rE7m/CpG, or rF9R/rE7m/CpG (**Figures 5A**, left). Seven days after the second immunization, splenocytes were harvested and stimulated for 48 hours with either RAH (HPV E7_49−57_) or a control peptide (OVA_257−264_), both of which are CD8^+^ T cell epitopes. The frequency of IFN-γ-secreting cells was then measured using an ELISPOT assay. Among the groups, the rF9R/rE7m/CpG formulation induced a significantly higher frequency of IFN-γ-producing CD8^+^ T cells compared to rE7m, rF9R/rE7m, or rE7m/CpG (**Figures 5A**, right). In contrast, negligible IFN-γ secretion was observed in response to OVA_257−264_, confirming the specificity of the immune response. These results indicate that the rF9R complex-based vaccine effectively enhances antigen-specific CD8^+^ T cell activation.

To assess the antitumor efficacy of these formulations, C57BL/6 mice were subcutaneously inoculated with TC-1 cells (2 × 10^5^) and treated on days 7 and 14 with PBS, rE7m, rF9R/rE7m, rE7m/CpG or rF9R/rE7m/CpG (**Figures 5B**, top). Tumor growth was monitored over time, revealing that while rF9R/rE7m and rE7m/CpG showed moderate tumor suppression, the rF9R/rE7m/CpG combination induced the most tumor reduction compared to PBS-treated controls (**Figures 5B**, bottom). These results suggest that the combination of rF9R with rE7m and CpG synergistically enhances the antitumor immune response.

To further elucidate the role of T-cell subsets in mediating this effect, mice were immunized and then selectively depleted of CD4^+^, CD8^+^, or isotype control antibodies prior to tumor implantation (**Figures 5C**, top). Monitoring of tumor growth revealed that depletion of CD8^+^ T cells completely abrogated the antitumor effect, whereas depletion of CD4^+^ T cells had certain impact (**Figures 5C**, bottom). This confirms that CD8^+^ T cells play a critical role in the antitumor efficacy of the rF9R-based vaccine.

In conclusion, these findings demonstrate that the rF9R complex, in combination with the antigen rE7m and the immunopotentiator CpG, robustly induces antigen-specific CD8^+^ T-cell responses and effectively suppresses tumor growth. This study supports further development of rF9R complex-based formulations as a promising strategy for cancer immunotherapy.

## 4 Discussion

This study explores the modification of the immunopotentiator rF9R by introducing the positively charged residues to enhance its association strength with anionic vaccine candidates, thereby improving immunogenicity. Our findings confirm that rF9R maintains strong binding affinity to both mouse and human Fcγ receptors, preserving its biological activity and therapeutic potential. This supports our hypothesis that rF9R functions as a versatile carrier and could facilitate vaccine development. Moreover, rF9R exhibits flexibility by binding to synthetic peptides, recombinant antigens, and immune-stimulatory molecules such as CpG. Previously, we demonstrated that antigen-FLIPr fusion proteins can effectively deliver antigens to DCs, inducing memory T cell responses and broad-spectrum cytokine production (11, 12). Similarly, the broad Fcγ receptor binding capacity of rF9R enables it to act as an efficient carrier for therapeutic agents. It can be incorporated into peptide and subunit vaccine formulations through simple mixing with antigen candidates, thereby streamlining the development of cancer vaccines.

Many delivery platforms, such as cationic liposomes, polymers, and cell-penetrating peptides, utilize electrostatic interactions to enhance antigen uptake. However, these systems often lack immune-targeting specificity and require complex chemical conjugation or formulation strategies (19, 20). In contrast, rF9R provides a dual-function mechanism that combines broad electrostatic binding capacity with targeted immune delivery. By engineering nine additional arginine residues into FLIPr, rF9R achieves a significantly increased net positive charge (+12.56 at pH 7.0 vs. +3.5 for native FLIPr), enhancing its affinity for negatively charged molecules such as peptides, protein antigens, and CpG oligonucleotides. Importantly, rF9R retains its ability to bind Fcγ receptors on antigen-presenting cells (APCs), enabling receptor-mediated endocytosis and facilitating antigen presentation through both MHC class I and II pathways-functions not typically achieved by conventional electrostatic carriers (11). This enhanced binding capability allowed rF9R to stably interact with highly anionic molecules, including 5D-LOT1 (–7.99), rE7m (–13.07), and CpG 1826 (–23.1), which showed minimal affinity for unmodified FLIPr (**Figure 2A**, **3A-B**, **4B-C**). These improvements in electrostatic interaction correlated with increased immunogenicity and antitumor efficacy in murine models.

In the EG7 tumor model, rF9R enhances synthetic peptide binding, leading to effective tumor suppression. The highest efficacy is observed when rF9R is combined with 5D-LOT1 and 5D-LOT2, which contain CD8 and CD4 epitopes, respectively. These findings suggest that CD4^+^ T cells are essential for eliciting robust CD8^+^ T cell responses. Similarly, in the TC-1 tumor model, rF9R combined with 5D-LRAH (containing a CD8 epitope) and 5D-Thp (containing a CD4 epitope) achieves greater tumor suppression than single-component treatments, underscoring the broader potential of this combinatorial approach. The cellular mechanisms underlying CD4^+^ T cell-mediated support for CD8^+^ T cell responses begin as early as DC priming of CD8^+^ T cells (21, 22). Multiple studies have demonstrated that CD4^+^ T cells are necessary for the induction and maintenance of robust CD8^+^ T cell responses (23–33). These findings highlight the critical role of CD4^+^ T cells across multiple stages of the CD8^+^ T cell response and may inform the design of more effective peptide-based vaccine strategies.

An important discovery of this study is that the rF9R/rE7m/CpG formulation induces robust CD8^+^ T-cell responses and considerable tumor growth inhibition in the TC-1 tumor model. The enhanced immune activation observed in this combination therapy underscores the critical role of rF9R in amplifying antigen-specific immune responses. The study reveals that this formulation significantly increases IFN-γ-secreting CD8^+^ T cells, indicating a heightened CTL response against tumor cells. Given the critical role of CD8^+^ T cells in recognizing and eliminating malignant cells, these findings highlight the potential of rF9R in cancer immunotherapy.

The production of rF9R follows well-established molecular biology techniques, ensuring a scalable, reproducible, and high-purity process. Subunit vaccines derived from molecular biology offer advantages such as enhanced safety, rapid production, and adaptability to emerging variants, making them highly effective for disease control. These attributes position rF9R as a promising candidate for both research and clinical applications.

While this study provides valuable insights using murine tumor models (EG7 and TC-1), these models may not fully capture the complexity of human tumor biology, including variations in genetic composition, tumor microenvironment, and immune interactions. As such, careful consideration is needed when relating these findings to human patients. Future research incorporating human xenograft models could help bridge this gap by offering a more representative system for studying human tumor biology and treatment responses. Additionally, while the study demonstrates promising short-term tumor suppression, further investigation into long-term outcomes, such as tumor recurrence and immune memory, would be highly beneficial. Understanding the durability of the antitumor response could provide deeper insights into the potential of rF9R-based therapies in preventing cancer relapse and enhancing long-term efficacy.

This study also underscores the important role of CD8^+^ T cells in antitumor immunity. Further research exploring the contributions of other immune cell populations, such as DCs, natural killer cells, and regulatory T cells, could offer a more comprehensive perspective on immune system dynamics. Additionally, more in-depth mechanistic studies could help clarify the molecular pathways involved in rF9R-mediated immune activation, including the downstream effects of Fcγ receptor interactions, the role of TLR9 in rF9R/CpG complex internalization, and the influence of rF9R on antigen presentation and cross-presentation by DCs.

The physicochemical properties of the complex, particularly particle size and surface charge (zeta potential), are known to significantly influence biological behaviors such as cellular uptake and systemic distribution (34, 35). Therefore, characterizing these parameters will be a priority in our future studies to further elucidate the underlying mechanisms and optimize the efficacy of the rF9R platform. In parallel with physical properties, the biological interaction between the vaccine and innate immune cells is equally critical. *In vitro* studies using macrophages are indispensable for developing effective cancer vaccines, as these cells play a central role in orchestrating innate immune responses within the tumor microenvironment (36). Macrophages exhibit remarkable plasticity and are often skewed toward an immunosuppressive, pro-tumor M2 phenotype (37). Therefore, a key objective in vaccine design is to reprogram them into a pro-inflammatory, tumoricidal M1 state (38). *In vitro* systems provide a controlled platform to evaluate whether vaccine components—such as adjuvants and delivery systems—can effectively drive this phenotypic shift. These models also enable precise quantification of cytokines (e.g., IL-12 and TNF-α) critical for initiating robust downstream T-cell responses (39). Moreover, high-throughput *in vitro* assays allow for the rapid screening of antigen immunogenicity and assessment of phagocytic activity, accelerating the optimization of vaccine formulations prior to *in vivo* studies (40). Collectively, these studies serve as a crucial bridge between rational vaccine design and functional immunogenicity in cancer immunotherapy.

In summary, this study presents compelling evidence for the therapeutic potential of rF9R in cancer immunotherapy. Its strengths lie in its rigorous experimental design, clear demonstration of efficacy, and potential for clinical relevance. While this study primarily focuses on specific tumor models and key immune mechanisms, future research expanding these aspects will further strengthen the clinical translation of rF9R-based therapies. Overall, these findings provide a strong foundation for further research and development in this promising area of cancer immunotherapy.

## Data Availability

The original contributions presented in the study are included in the article/supplementary material. Further inquiries can be directed to the corresponding authors.
